# Analysis of a Set of KDM5C Regulatory Genes Mutated in Neurodevelopmental Disorders Identifies Temporal Coexpression Brain Signatures

**DOI:** 10.3390/genes12071088

**Published:** 2021-07-18

**Authors:** Loredana Poeta, Agnese Padula, Maria Brigida Lioi, Hans van Bokhoven, Maria Giuseppina Miano

**Affiliations:** 1 Institute of Genetics and Biophysics Adriano Buzzati-Traverso, CNR, 80131 Naples, Italy; agne-88@hotmail.it; 2 Department of Science, University of Basilicata, 85100 Potenza, Italy; maria.lioi@unibas.it; 3 Department of Human Genetics, Donders Institute for Brain, Cognition and Behaviour, Radboudumc, 6525 GA Nijmegen, The Netherlands; Hans.vanBokhoven@radboudumc.nl

**Keywords:** neurodevelopmental disorders, X-chromosome, KDM5C, H3K4me3, disease mutations, NDD-related chromatinopathies

## Abstract

Dysregulation of transcriptional pathways is observed in multiple forms of neurodevelopmental disorders (NDDs), such as intellectual disability (ID), epilepsy and autism spectrum disorder (ASD). We previously demonstrated that the NDD genes encoding lysine-specific demethylase 5C (*KDM5C*) and its transcriptional regulators Aristaless related-homeobox (ARX), PHD Finger Protein 8 (PHF8) and Zinc Finger Protein 711 (*ZNF711*) are functionally connected. Here, we show their relation to each other with respect to the expression levels in human and mouse datasets and in vivo mouse analysis indicating that the coexpression of these syntenic X-chromosomal genes is temporally regulated in brain areas and cellular sub-types. In co-immunoprecipitation assays, we found that the homeotic transcription factor ARX interacts with the histone demethylase PHF8, indicating that this transcriptional axis is highly intersected. Furthermore, the functional impact of pathogenic mutations of *ARX*, *KDM5C*, *PHF8* and *ZNF711* was tested in lymphoblastoid cell lines (LCLs) derived from children with varying levels of syndromic ID establishing the direct correlation between defects in the KDM5C-H3K4me3 pathway and ID severity. These findings reveal novel insights into epigenetic processes underpinning NDD pathogenesis and provide new avenues for assessing developmental timing and critical windows for potential treatments.

## 1. Introduction

Brain development and neuronal differentiation are complex processes involving spatio-temporal changes in chromatin remodeling and transcriptional activity in specific brain regions and neural cell types [1,2,3]. Alterations in regulatory genes of chromatin remodeling and transcription factors (TFs) cause a huge spectrum of neurodevelopmental disorders (NDDs) [2,4,5,6]. We recently uncovered genetic and functional relationships within a set of X-linked NDD genes formed by the epigenetic eraser Lysine-Specific Demethylase 5C (*KDM5C*; MIM:314690) and its upstream regulators, Aristaless-related homeobox (*ARX*; MIM:300382), PHD Finger protein 8 (*PHF8*; MIM:300560) and Zinc Finger protein 711 (*ZNF711*; MIM:314990) [4,5]. *KDM5C* encodes a highly conserved JmjC-domain protein that reverts the H3K4me3 substrate into di- and mono-methylated products, acting as a fine-tuning transcriptional regulator essential for dendritic spine plasticity [7,8,9]. Mutations in *KDM5C* were generally found in male children with X-linked syndromic Intellectual Disability (XLID) Claes-Jensen type (MIM:300534), characterized by moderate to severe ID, spasticity, epileptic seizures, short stature, and microcephaly [10,11,12] or showing a developmental delay with or without ASD-like signs [13]. Point mutations or defective gene expression of *KDM5C* may compromise its demethylase activity leading to target gene expression deregulation [5,14]. *ARX* encodes a homeotic bi-functional TF capable of activating or repressing gene transcription [15]. Mutations in *ARX* have been found in a wide range of NDDs, affecting only male children and including severe cortical malformations such as X-linked Lissencephaly with agenesis of the corpus callosum and ambiguous genitalia (XLAG; MIM:300215); Agenesis of Corpus Callosum (ACC; MIM 300004; also known as Proud syndrome); severe pediatric epilepsy such as Developmental and Epileptic Encephalopathy 1 (DEE1; MIM:308350, also known as West syndrome); and mild cognition diseases including Partington syndrome (MIM 309510), autism, and non-syndromic intellectual disability [4,16,17,18,19,20]. Previous studies conducted by us and other groups have shown that XLAG mutations abrogate the transcriptional program controlled by ARX, whereas expansions of the polyA-tracts are hypomorphic mutations with reduced transcriptional activity and binding properties [4,5,15,21,22]. *PHF8* encodes a histone demethylase whose mutations were found in male children patients with Siderius X-linked mental retardation syndrome (MRXSSD; MIM:300263) characterized by ID, microcephaly, and cleft lip and palate [23,24,25]. Finally, *ZNF711* encodes a TF of the Krüppel C2H2-type zinc-finger protein family that binds mainly to GC-rich CpG island promoters [26]. Variants in *ZNF711* have been found in children with non-syndromic ID (MIM:300803) presenting mild to moderate cognition defect and poor speech accompanied by autistic features and mild facial dysmorphisms in some patients [27,28,29]. Of interest, both *KDM5C* and its transcriptional regulatory genes are dispersed across the X-chromosome that has been historically targeted for ID studies because variants in these genes give rise to familial recurrence of cognitive defects that affect males more often and more severely than females [30]. Although we have some knowledge of the intersecting regulation of this set of NDD genes, little is currently known about their transcriptional trajectories in the developing brain [5,31]. Consequently, there is no information on their relation to each other with respect to the expression profiles in specific brain areas and/or neuronal cells. To fill this gap, we here report on where and when *KDM5C* and its three regulatory genes are coexpressed. To this end, we integrated data available in public human and mouse databases with in vivo studies performed on embryonic and neonatal mouse brains. Additionally, in NDD patient-derived lymphoblastoid cell lines (LCLs) mutated in *KDM5C* and in its regulatory genes, we also figure out the functional correlation between gene mutation severity and the defect in the KDM5C-H3K4m3 pathway.

## 2. Materials and Methods

### 2.1. Animals

Protocols for animals were approved by the Italian Ministry of Health (DLgs116/92) in accordance with the Institutional Animal Care guidelines of the Institute of Genetics and Biophysics Adriano Buzzati-Traverso.

### 2.2. Identification of Cap Analysis of Gene Expression (CAGE) Derived Transcription Start Sites

Cap Analysis of Gene Expression (CAGE) is a powerful experimental technique for assisting in the identification of transcription start sites (TSSs;) [32,33]. The FANTOM Consortium2 has extensively applied CAGE on hundreds of tissues and cell lines of humans and mice and characterized the regulatory mechanisms of gene expression. Analysis of FANTOM5 database was performed using Zenbu browser genomic data visualization tools (https://fantom.gsc.riken.jp/zenbu/ accessed on 1 May 2021). Robust CAGEs defined by DPI clustering falling inside the RefSeq regions of *KDM5C*, *ARX*, *PHF8* and *ZNF711* genes were selected. The expression values are shown in Tags Per Million (TPM) calculated on a per-library total expression. TPM values were obtained for each CAGE using the FANTOM5 expression dataset for tissues, cell lines, and primary cells in human and mouse (Supplementary Tables S1 and S2).

### 2.3. Cell Lines, Transient Transfection, and Luciferase Assay

SH-SY5Y cell line was maintained in Dulbecco’s modified Eagle’s medium (Life Technologies™ Scotland, Swindon, UK) supplemented with 10% fetal bovine serum (FBS, Life Technologies™ Bleiswijk, Bleiswijk, The Netherlands), 100 units/mL penicillin and 100 mg/mL streptomycin (Gibco BRL, Paisley, Scotland). For lymphoblastoid cell line (LCL) establishment, peripheral blood mononuclear cells (PBMCs) were isolated and cultured in RPMI 1640 (Life Technologies, Swindon, UK) using standard methods. All cell lines were tested regularly for mycoplasma contamination.

Transient transfections were performed according to standard methods [4]. The reporter activities were measured using the Dual-Luciferase Reporter Assay System (Promega Corporation Madison, Madison, WI, USA). Each assay was performed in duplicate in three independent experiments and the resulting firefly luciferase values were normalized using the renilla values.

### 2.4. Plasmids

The wild-type *ARX* expression plasmid and its mutants c.298_330dup33 [p.Ala105_Ala115] and c.429_452dup24 [p.Ala148_Ala155] have been previously described [4]. The pCMV-HA tagged *PHF8*, pCMV-Myc tagged *PHF8*, and pCMV-Myc tagged *ZNF711* constructs expressing the human cDNA of *PHF8* and *ZNF711* are a gift from K. Helin (Center for Epigenetics Research at Memorial Sloan Kettering Cancer Center & University of Copenhagen, København, Denmark) described elsewhere [34]. Immunoblot analysis of the transfected cell lysates was performed to prove that the overexpressed protein levels were present in all cell lines.

### 2.5. Western Blotting and Protein Interaction Assays

Protein extracts from mammalian cells and tissues were prepared and separated following standard methods [5]. After the blocking of nonspecific binding sites on the membranes with 5% non-fat milk, the membranes were incubated with specific antibodies. The following antibodies were used: anti-ARX (2 μg/mL final; H70 sc-98895, Santa Cruz Biotechnology, Inc. Europe, Heidelberg, Germany), anti-KDM5C (1:1000, H-99 sc-98701, Santa Cruz Biotechnology, Inc. Europe, Heidelberg, Germany), anti-H3K4me3 (1:5000, AB-8580, Abcam Cambridge, Waltham, MA, USA), anti-PHF8 (1:500, Millipore 09-868, Merck, Darmstadt, Germany), anti-SYN1 (1:300, Calbiochem 574777, Merck, Darmstadt, Germany), anti-ZNF711 (1:1000, AB-187324, Abcam Cambridge, Waltham, MA, USA). Anti-β-actin (1:3000, C4 sc-47778, Santa Cruz Biotechnology, Inc. Europe, Heidelberg, Germany) was used as loading controls. The signals were detected with an enhanced chemiluminescence kit (Amersham Biosciences, British Isles, Buckinghamshire, UK) and the films were processed for densitometric scanning. The protein interaction studies were performed using Dynabeads^®^ Protein G Immunoprecipitation Kit (Thermo Fisher Scientific, Waltham, MA, USA) following the manufacturer’s instructions. For the immunoprecipitation assays of the endogenous PHF8 and ARX, total lysates from SH-SY5Y cells were used. After SDS-page, Western blotting was performed using anti-ARX (1:500, H70 sc-98895, Santa Cruz Biotechnology, Inc. Europe, Heidelberg, Germany) and anti-PHF8 (1:500, Millipore 09-868, Merck, Darmstadt, Germany) antibodies. For the co-immunoprecipitation assays, the SH-SY5Y cells were transfected with the expression vectors pCMV-HA-PHF8, pCMV-Myc-ZNF711 and pCMV-Myc-ARX. The antibodies used were the anti-cMyc antibody (1:5000, Clone 9E10 M4439, Sigma, Saint Louis, MO, USA) and anti-HA antibody (1:5000, AB-9110, Abcam Cambridge, Waltham, MA, USA). The flow-through derived from each experiment was tested as a negative control of the binding specificity.

### 2.6. RNA Extraction and Real-Time Polymerase Chain Reaction

Total RNA extraction was carried out with TRIzol procedure (Life Technologies, Scotland, Swindo, UK). Reverse transcription was performed with a QuantiTect Reverse Transcription kit (Qiagen, Hilden, Germany) and steady-state mRNA abundance was determined using the Power SYBR Green PCR Master Mix (Applied Biosystems, Foster City, CA, USA) on the 7900HT Fast Real-Time PCR System (Applied Biosystems, Foster City, CA, USA). Sequences of oligonucleotide primers used in qRT-PCR are reported in Supplementary Table S3. Each experimental assay was performed in triplicate in three independent experiments.

### 2.7. Statistical Analysis

The statistical analyses were performed with the GraphPad Prism 4 software (GraphPad Software) to calculate the significance of the differences among each sample against the control. One-way ANOVA test with Bonferroni correction or the Student’s *t*-test was used. The standard error of the mean was used to estimate variation within a single assay.

## 3. Results

### 3.1. Spatiotemporal Expression Patterns of KDM5C and Its Regulatory Genes in Human and Mouse Brain

**Figure 1:**
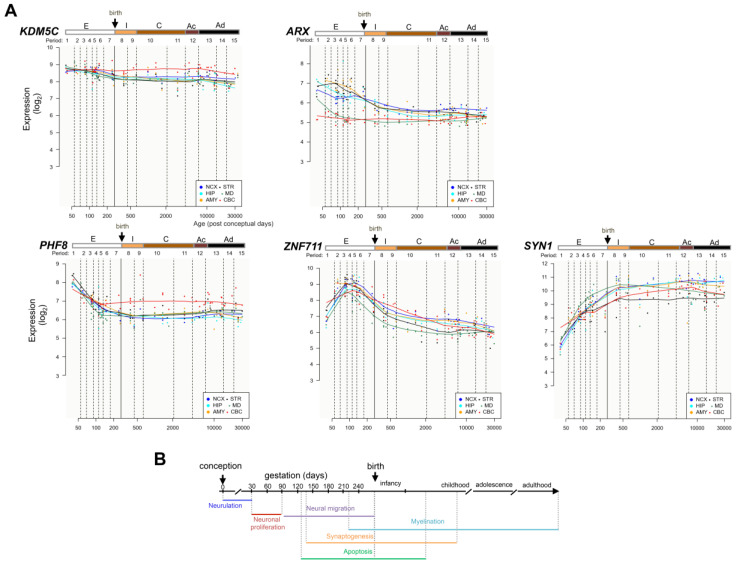
Expression profiles of *KDM5C*, *ARX*, *PHF8*, and *ZNF711* during development and in the adult human brain. (**A**) Data from Human Brain Transcriptome Atlas are shown in RPKM. Original images can be accessed freely at https://hbatlas.org/hbtd/basicSearch.pl (accessed on 2 April 2021). Period 1, 4 < Age < 8 postconceptional weeks (PCW); Period 2, 8 < Age < 10 PCW; Period 3, 10 < Age < 13 PCW; Period 4, 13 < Age < 16 PCW; Period 5, 16 < Age < 19 PCW; Period 6, 19 < Age < 24 PCW; Period 7, 24 < Age < 38 PCW; Period 8, Neonatal and early infancy, birth ≤ Age < 6 postnatal months; Period 9, Late infancy, 6 < Age < 12 postnatal months; Period 10, Early childhood, 1 < Age < 6 years; Period 11, Middle and late childhood, 6 < Age < 12 years; Period 12, Adolescence, 12 < Age < 20 years; Period 13, Young adulthood, 20 < Age < 40 years; Period 14, Middle adulthood, 40 < Age < 60 years; Period 15, Late adulthood, 60 + years [35]. Embryonic development (E), from 1 to 7; birth and infancy (I), from 8 to 9; childhood (C), from 10 to 11; adolescence (Ac), 12; adulthood (Ad), from 13 to 15. Brain regions: neocortex (NCX), hippocampal formation (HIP), amygdala (AMY), striatum (STR), midbrain (MB), cerebellum (CBC). (**B**) A glimpse of developmental-stage-specific chronology for neurogenesis (from the embryonic life to the adult stage in human).

To test whether *KDM5C* and its three regulator genes are coexpressed across embryonic and adult brain tissues, we used public resources of the Human Brain Transcriptome Atlas available at https://hbatlas.org/hbtd/basicSearch.pl (accessed on 2 April 2021) [35]. In six brain areas—neocortex (NCX), hippocampal formation (HIP), amygdala (AMY), striatum (STR), midbrain (MB), and cerebellum (CBC)—we observed the high expression levels of the two erasers *KDM5C* and *PHF8* in the early days of neuronal development (period 1: embryonic development 4 < Age < 8 PCW corresponding to the first lamination of the cerebral wall; Figure 1A,B). Then, we observed a progressively declined decrease with age until the birth (period 7: late fetal development 24 < Age < 38 PCW characterized by the transition from the typical fetal lamination pattern into an adult-like lamination pattern of the cerebral wall; Figure 1A,B). The transcription factor genes *ARX* and *ZNF711* showed a progressive increase in the early stages of development until period 3 (early fetal development 10 < Age < 13 PCW characterized by the presence of the bilaminate cortical plate) and period 4 (early mid-fetal development 13 < Age < 16 PCW characterized by the consolidation of the cortical plate and the formation of a large, synapse-rich subplate zone). In particular, *ARX* showed an oscillator expression profile exhibiting an increase in STR (period 3; Figure 1A,B), followed by a change in the opposite direction, with a progressive decreasing trend in the remaining five brain areas, from the early days of neuronal development to the birth (Figure 1A,B). Regarding *ZNF711*, its gene expression increased gradually from the first days of neuronal development with a peak at the middle of the embryonic phase and then gradually decreased until birth (period 3; Figure 1A,B). Additional evidence for their coexpression during neuronal differentiation derives from transcriptomics resources of human induced pluripotent stem cells (hiPSC) differentiated into neurons (Supplementary Figure S1; http://stemcell.libd.org/scb/ accessed on 1 August 2021). Furthermore, in the postnatal lifespan, we observed a similar trend for each NDD gene among the regional expression profiles in human brain (Figure 1A). In addition, we analyzed the expression profile of the X-linked epilepsy gene Synapsin I (*SYN1;* MIM:313440; [36]), a repressed downstream target of *KDM5C* encoding a neuronal phosphoprotein involved in synaptic neurotransmission [5]. In agreement with this regulation pattern, we observed a gradual increase of *SYN1* in each of the six brain areas, from the early days of conception to the birth (Figure 1A).

**Figure 2:**
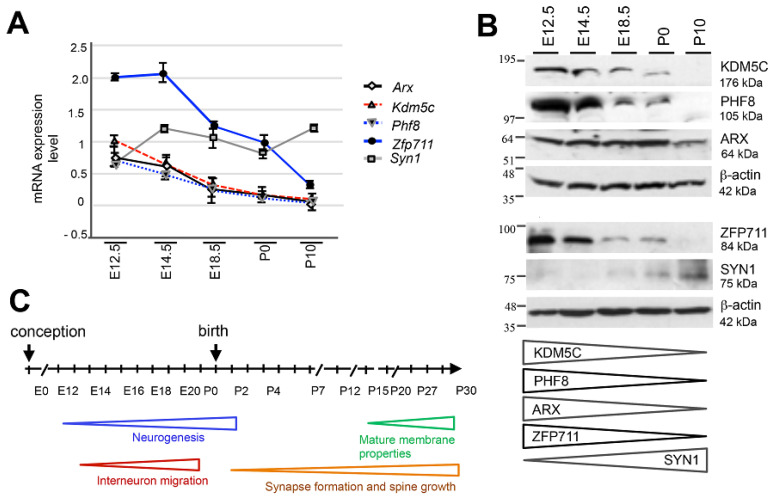
Expression profiles of *Kdm5c*, *Arx*, *Phf8* and *Zfp711* in embryonic and neonatal mouse brain. Representative quantitative real-time PCR (**A**) and western blot analysis (**B**) of *Arx*/ARX, *Phf8*/PHF8, *Kdm5c*/KDM5C, *Syn1*/SYN1 and *Zfp711*/ZFP711, throughout prenatal and postnatal stages in the whole WT murine brain. The transcript analysis was performed in triplicate and the samples were normalized to *18S*. The bars indicate the mean ± standard error of three independent experiments. The Western blotting experiment was repeated three times. The beta-actin antibody (β-actin) was used as a loading control. (**C**) Schematic representation of the neuronal developmental time line (days) from conception to birth and from birth until 30 days after birth (P30).

Next, we assessed the dynamics of this regulatory axis during mouse embryonic development (at E12.5, E14.5, and E18.5) and during post-natal life (at birth, P0, and P10; Figure 2A–C). By real-time PCR and western blot analysis, we observed that *Kdm5c* (GenBank NM_145997.2), *Arx* (GenBank: BC052033.1), *Znf711* (in mouse named *Zfp711*, GenBank: NM_177747.3), and *Phf8* (GenBank: BC138899.1) are all highly expressed at the beginning of neurogenesis (between E12.5 and E14.5), both at mRNA and protein levels (Figure 2A–C). Then, the level of *Kdm5c*/KDM5C gradually decreases during synapse formation (from E18.5 to P10; Figure 2A–C), as well as *Zfp711*/ZFP711 and *Phf8*/PHF8. Regarding the level of *Arx*/ARX, we observed a gradual decrease from E18.5 to P10 as mRNA and a similar protein band from E12.5 to P0 that declines after birth (Figure 2A,B). Taken together, these data provide evidence that the expression patterns of *Kdm5c* and its regulatory genes overlap prenatally and disappear post-natally. Moreover, in situ hybridization images obtained from the Allen Institute for Brain Science showed that in adult male mice, *Kdm5c, Arx, Phf8*, and *Zfp711* are coexpressed mainly in the hippocampus and olfactory bulbs (Supplementary Figure S2; https://mouse.brain-map.org/ accessed on 8 April 2021). Noteworthily, the hippocampus and olfactory bulbs are the sites of adult neurogenesis [37]. We also tested the level of *Syn1*, which transcriptional expression is known to be repressed by KDM5C [38,39]. As expected, the SYN1 protein displays a later onset (E18.5) followed by a gradual increase after birth, which inversely mirrors the decrease of KDM5C (Figure 2B). In conclusion, these findings highlight that in both human and mouse, the *KDM5C/Kdm5c* transcriptional regulation is organized into coexpression modules operating at specific timing of neurogenesis.

### 3.2. CAGE-Defined TSSs of KDM5C and Its Regulatory Genes in Human and Mouse Brain

**Figure 3:**
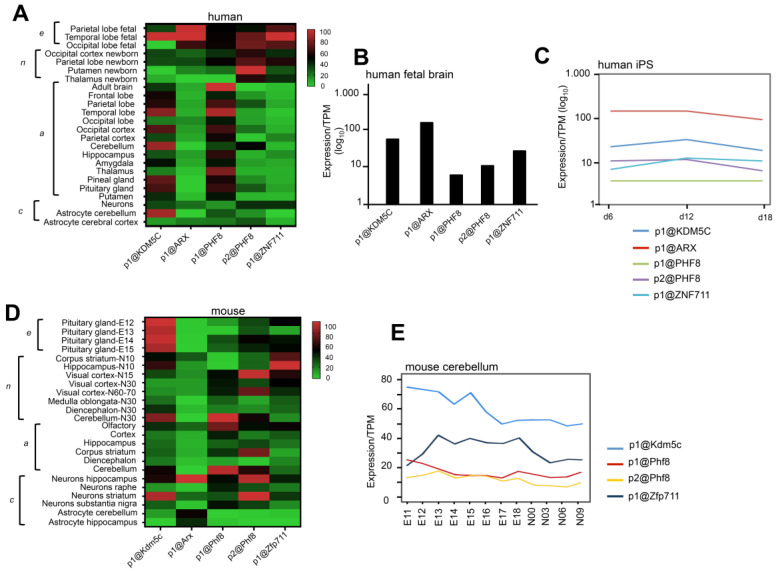
Expression profiles of TSSs in human (**A**–**C**) and mouse (**D**,**E**) brain samples. Heat map showing the TPM expression of all promoters in sub-regions of brains and brain-related primary cells in human (**A**). Expression profiles of p1@KDM5C, p1@ARX, p1@PHF8, p2@PHF8, and p1@ZNF711 in human fetal brain (**B**) and in human iPS differentiated to neuron (**C**), (day 6 = neuro-ectodermal cells; day 12 = neural stem cells; day 18 = early neuronal progenitors). The expression of each CAGE promoter is reported as TPM on the *y*-axis. Heatmap showing the TPM expression of all promoters in sub-regions of brains and brain-related primary cells in mice (**D**). Expression profiles of p1@Kdm5c, p1@Arx, p1@Phf8, p2@Phf8, and p1@Zfp711 in mouse cerebellum (**E**). Heatmap’s data are normalized and are reported as percentages with respect to the largest value in each data set. Data are available at https://fantom.gsc.riken.jp (accessed on 1 May 2021).

We analyzed data of promoter level expression across the transcriptome of different brain areas and neuronal cells from the FANTOM 5 project database [40] (https://fantom.gsc.riken.jp/zenbu/ accessed on 1 May 2021). By using the Cap Analysis of Gene Expression method (CAGE), we identified the predominantly used transcription start sites (TSSs) for the four genes *KDM5C*, *ARX*, *PHF8*, and *ZNF711* (Supplementary Figure S3 and Table S1). Specifically, we established that in human brain, the highest used TSS for *KDM5C* is p1@KDM5C located at chrX:53211978-53263129(-); for *ARX*, it is p1@ARX located at chrX:25034067-25034088(-); for *PHF8*, we identified p1@PHF8, located at chrX:54070731-54070781(-), and p2@PHF8 located at chrX:54070879-54070917(-); and for *ZNF711*, it is p1@ZNF711 located at chrX:84498989-84499003(+) (Supplementary Figure S3A–C and Table S1). In mouse brain, the highest used *Kdm5c* TSS is p1@Kdm5c located at chrX:148667771-148667803(+); for *Arx*, it is p1@Arx located at chrX:90531838-90531861(+); for *Phf8*, it is p1@Phf8, located at chrX:147955689-147955749(+), and p2@Phf8 located at chrX:147955198-147955224(+); and for *Zfp711*, it is p1@Zfp711 located at chrX:109714331-109714347(+) (Supplementary Figure S3A–C and Table S1). Successively, we extracted the expression value for each TSS measured as tag per million (TPM) (Figure 3A–C). We generated the heatmap of human promoters using the value of TPM expression (Figure 3A; Supplementary Table S2). We found a strong expression of p1@KDM5C, p1@ARX, p1@PHF8, p2@PHF8 and p1@ZNF711 (expression module embryonic-*e*) in fetal sub-areas of the cortex (Figure 3A, panel *e*). In line with these evidences, the TSSs of the four NDD genes are expressed in a fetal brain pool (59 samples between 20-33 weeks; CNhs1086459—from p1@ARX, the most expressed, to p1@PHF8, the least expressed (Figure 3B; Supplementary Table S2). Moving to neonatal and adult brain samples, we found several changes with respect to fetal profiles (Figure 3A, panels *n* and *a*). In the neonatal brain samples, the coexpression of the TSSs is still present but less strong (expression module neonatal-*n*). Instead, in the adult samples, we observed a robust coexpression only of p1@KDM5C and p2@PHF8 since the levels of p1@ARX and p1@ZNF711 are severely reduced in most of the adult samples (expression module adult-*a*; Figure 3A, panel *a*). More interestingly, primary neuronal cells showed a weak coexpression of p1@KDM5C, p1@ARX, p2@PHF8, and p1@ZNF711 (expression module-neuron cell-*c*; Figure 3A), while astrocytes showed two distinctive coexpression profiles: p1@KDM5C, p1@PHF8, and p2@PHF8 in cerebellum (expression module-astrocyte cerebellum-*c*; Figure 3A); and p1@KDM5C, p1@ARX, p1@PHF8, p2@PHF8, and p1@ZNF711 (expression module-astrocyte cortex-*c*; Figure 3A). Additionally, in neuronally differentiated induced pluripotent stem cells (iPS), we observed that p1@KDM5C, p1@ARX, p2@PHF8, and p1@ZNF711 are expressed from the induction of neuro-ectodermal cells (day 6) to the generation of early neuronal progenitors (day 18). They showed an oscillatory trend with a peak of p1@ARX and p2@PHF8 at day6 and of p1@KDM5C and p1@ZNF711 at day 12 corresponding to the generation of self-renewing multipotent neural stem cells (Figure 3C).

In neonatal mice, we found a strong expression of p1@Kdm5c, p1@Arx, p2@Phf8, and p1@Zfp711 in corpus striatum (STR) and hippocampus (HIP), both at age N10 (expression module STR/HIP-*n*) and in visual cortex at age N15 (Figure 3D and Supplementary Table S2). In adult mice, we noted a strong expression of all TSSs only in the olfactory area (expression module OP-*a*). In the remaining areas, a strong decrease in p1@Arx was observed in cortex (CX), HIP, and cerebellum (CB) with respect to OP (expression module CX/HIP/CB-*a;* Figure 3D). Moving to primary cells, we found strong expression of all TSSs in neurons isolated from HIP and STR (coexpression module HIP/STR-*c*; Figure 3D). Of note, p1@Arx is poorly expressed in neurons of the substantia nigra but strongly expressed in astrocytes of the CB and HIP. Moreover, in CB, the coexpression of p1@Kdm5c and p1@Zfp711 is evident, and the weak expression of p1@Phf8, p2@Phf8, while p1@Arx expression was not detected (Figure 3E; Supplementary Table S2). Collectively, the TSS data obtained both in human and mouse samples strongly support the hypothesis that *KDM5C/Kdm5c*, *ARX*/*Arx*, *ZNF711/Zfp711*, and *PHF8*/*Phf8* constitute a core regulatory network activated consistently in the same or distinctive cell type or brain areas in mammals.

### 3.3. KDM5C, ARX, PHF8, and ZNF711 Are Syntenic Genes of Human/Mouse X-Chromosome

**Figure 4:**
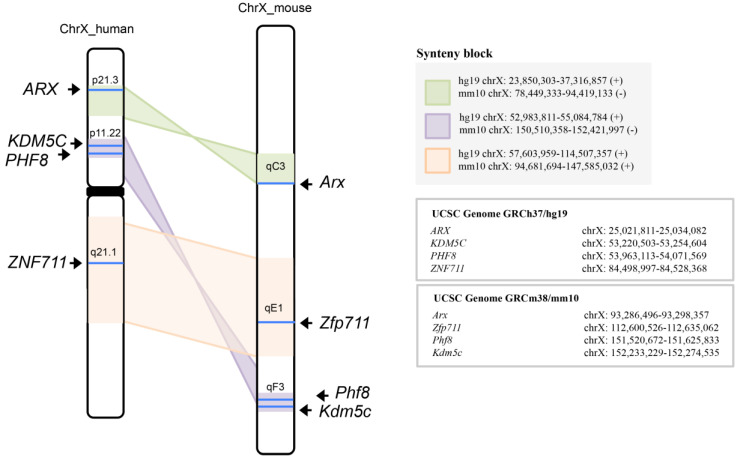
*KDM5C*, *ARX*, *PHF8*, and *ZNF711* are located in syntenic segments of human and mouse X chromosome (ChrX_ human and ChrX_mouse, respectively). SynBrowser map of the human and mouse X chromosome and the regions containing the genes (not to scale; http://bioinfo.konkuk.ac.kr/synteny_portal/, accessed on 1 May 2021)**.** Block linkages in the same orientation are labelled in pink (ChrX human Xq21.1 vs. ChrX mouse XqE1), while those in inverted orientation are labelled in green (ChrX human Xp21.3 vs. ChrX mouse XqC3) or in violet (ChrX human Xp11.22 vs. ChrX mouse XqF3). Nucleotide positions of each block are shown.

Gene order is not random with regard to gene expression in mammals but is maintained by natural selection because it has a functional significance [41]. We analyzed genome annotations of *KDM5C* and its regulatory genes on human (UCSC Genome GRCh37/hg19) and mouse (UCSC Genome GRCm38/mm10) genome assembly. As shown in Figure 4, the four genes *KDM5C, ARX, PHF8*, and *ZNF711* are all located in syntenic blocks of X chromosome. Specifically, the nearby genes *KDM5C* (hg19 chrX: 53,220,503–53,254,604) and *PHF8* (hg19 chrX: 53,963,113–54,071,569), at physical distance between them of 708 Kb—map to the region of human X chromosome p11.22. This region is syntenic to the inverted region of the mouse X chromosome qF3 where their murine counterparts, *Kdm5c* (mm10 chrX: 152,233,229–152,274,535) and *Phf8* (mm10 chrX: 151,520,672–151,625,833), map—physical distance between them is 607 Kbs. The human *ARX* (hg19 chrX: 25,021,811-25,034,082) maps to the Xp21.3 region syntenic to the inverted region of mouse X chromosome qC3 where maps the murine *Arx* orthologue (mm10 chrX: 93,286,496–93,298,357) maps; and finally, the human *ZNF711* (hg19 chrX: 84,498,997–84,528,368) maps to the Xq21.1 region syntenic to the mouse X chromosome qE1, where the murine counterpart named *Zfp711* maps (mm10 chrX: 112,600,526–112,635,062). Although it lacks an understanding of how these clusters may exist in mammals and what their functional significance is, we may assume that—due to their functional relationship during the brain development—KDM5C and its transcriptional regulators may tend to be clustered within the same chromosome.

### 3.4. ARX Interacts with PHF8

**Figure 5:**
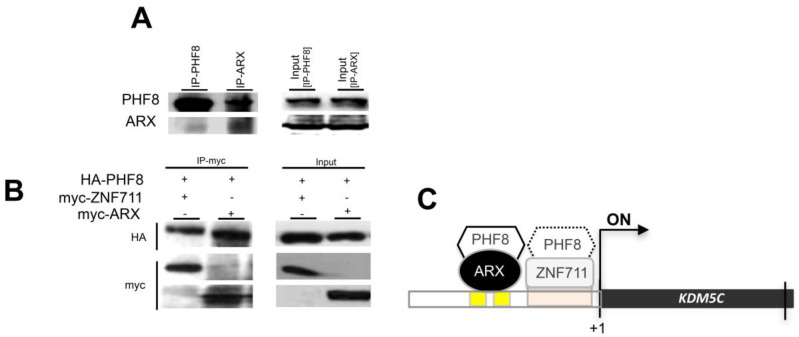
Co-immunoprecipitation of PHF8 and ARX in SH-SY5Y cells. Co-IP of endogenous PHF8 and ARX and vice versa. Cell lysates were subjected to immunoprecipitation with anti-PHF8 or anti-ARX antibodies. The presence of PHF8 and ARX in the cell extracts prior to immunoprecipitation was checked using anti-PHF8 and anti-ARX antibodies (Input) (**A**). Cells were transfected with a pCMV vector to express HA tagged-PHF8 (120 kDa), or Myc tagged-ARX (75 kDa), or Myc tagged-ZNF711 (100 kDa) (**B**). The cell lysates were subjected to immunoprecipitation with anti-HA or anti-Myc- antibodies. The presence of PHF8-HA, ZNF711-Myc, and ARX-Myc in cell extracts prior to immunoprecipitation was checked using anti-HA and anti-Myc antibodies (Input). Regulatory interactions with activating effects on *KDM5C* transcription are shown in the carton (**C**).

In a previous study, we showed that PHF8 and ARX acted cooperatively to induce KDM5C stimulation [5]. Therefore, we wondered whether this activity is mediated by a molecular interaction of PHF8 with ARX. To this end, we evaluated by co-immunoprecipitation (Co-IP) the ability of these regulatory transcriptional factors to interact directly in both endogenous and transiently transfected conditions. As shown in Figure 5A, endogenous ARX was immunoprecipitated by the anti-PHF8 antibody and vice versa, suggesting a direct protein-protein interaction. In addition, the formation of the ARX/PHF8 complex was observed in the coexpression of the HA- and Myc-tagged versions of *PHF8* and *ARX* (Figure 5B). The coexpression of the HA- and Myc-tagged version of *PHF8* and *ZNF711* was tested as the positive control of their direct interaction, as previously described [34] (Figure 5B). Thus, our results have revealed a direct connection between PHF8 and ARX, expanding the regulatory intersection of KDM5C stimulation (Figure 5C).

### 3.5. Analysis of KDM5C and H3K4me3 Levels in NDD Patient-Derived Lymphoblastoid Cell Lines

**Figure 6:**
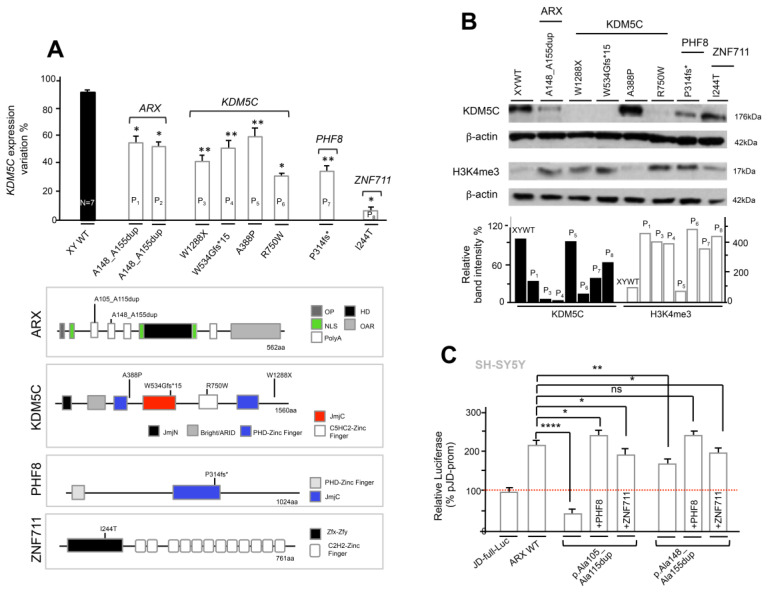
KDM5C defects associated to NDD mutations. KDM5C expression in lymphoblastoid cell lines obtained from distinct NDD patients mutated in *ARX, KDM5C, PHF8*, and *ZNF711.* The percentages indicate the expression variation with respect to the WT, which has a mean of seven (*n* = 7) different cell lines from male age-matched males. The transcript analysis was performed in triplicate and the samples were normalized to *18S*. The bars indicate the mean ± standard error of three independent experiments (*top panel*). Positions of the studied mutations are shown in the cartoon (*bottom panel*). OP, Octapeptide; polyA, polyalanine tracts; NLS, Nuclear localization sequences; HD, homeodomain; OAR, Aristaless domain; JmN, jumonji-N domain; ARID, AT-rich interacting domain; PHD, plant homeodomain box domain; JmjC, jumonji-C catalytic domain; ZF, zinc finger domain; PLU-1, PLU-1-like domain; Zfx/Zfy, Transcription activation domain (**A**). Western blotting for KDM5C and its substrate H3K4me3 was performed on cellular extracts from LCLs. The β-actin antibody (β-actin) was used as a loading control. Each experiment was repeated with two independent LCL protein extracts with similar results (**B**). Effects of PHF8 and ZNF711 overexpression on KDM5C 5′ UTR region in cotransfection with *ARX* polyalanine elongated mutants. Each luciferase assay was performed in duplicate in four independent experiments. The bars indicate the mean ± standard error of four independent experiments (**C**). * *p* < 0.05; ** *p* < 0.005; **** *p* < 0.0005; ns, not significant.

We set out to investigate on *KDM5C* expression and H3K4me3 levels in Epstein-Barr virus (EBV)-transformed lymphoblastoid cell lines (LCLs) obtained from eight NDD patients (P) with mutations in *KDM5C* or in its three regulators (Table 1). Specifically, the NDD P1 and P2 LCL cells were obtained from two unrelated male patients, presenting mild cognition defects, who carry the same in-frame *ARX* duplication c.429_452dup24 [p.A148_A155dup] (Figure 6A; [4,18,42,43]). From P3 to P6, LCLs were obtained from four male patients with mutations in *KDM5C* characterized by a variable degree of ID and comorbidities. Specifically, the nonsense mutations c.3864G>A [p.W1288X] [10,44] and c.1599delC [p.W534Gfs*15] [45] from patients P3 and P4, respectively, are both characterized by severe ID with epilepsy (Table 1); the missense mutation c.1162G>C [p.A388P] [10] in patient P5, presents ID with mild facial dysmorphism (Table 1); and the missense mutation c.2248C>T [p.R750W] [46] in patient P6, presents severe ID and speech impairment (Table 1). P7 LCLs were obtained by a male patient with ID and cleft/lip palate who carries the nonsense mutation c.1050_1061del12 [p.P314fs*] in *PHF8* [23] (Table 1). Finally, P8 LCLs were obtained from a male patient with mild ID who carries the missense mutation c.731T>C [p.I244T] in *ZNF711* [29] (Table 1).

By real-time PCR, we firstly detected a variable expression of the *KDM5C* mRNA in patient-derived LCLs with respect to the control male-derived LCLs (Figure 6A): in the *ARX*, P1 and P2 patients expressed 57% and 56% lower compared with the WT controls, respectively; in *KDM5C*, P3-P6 expressed 41%, 47%, 60%, and 29%, respectively; in *PHF8*, P7 expressed 31%; and in *ZNF711*, P8 expressed 5%. Secondly, by Western blotting we tested the protein levels of KDM5C and of its main substrate trimethylation of lysine 4 of histone H3 (H3K4me3). In line with the real-time-PCR data, we found that all mutations in *ARX, PHF8*, and *ZNF711* lead to a decrease in KDM5C protein content (Figure 6B). This defect inversely correlates with an increase in the H3K4me3 level, potentially as the result of the compromised demethylase activity of KDM5C (Figure 6B). These findings suggest that the mutations in *KDM5C* regulators act on the KDM5C-H3K4m3 pathway as partial loss-of-function because of a variable basal activity depending on the type of mutations. About *KDM5C* mutations, the nonsense *KDM5C* p.W1288X and *KDM5C* p.W534Gfs*15 mutations operate as loss-of-function mutations since they are incapable of synthesizing the relative proteins, and thus, of demethylase H3K4me3 (Figure 6B). Noteworthily, the missense mutation *KDM5C* p.A388P, showed a protein intensity band of KDM5C and H3K4me3 similar to that of the WT. In line with the predicted impact on the protein conformation reported previously [47], we conclude that this missense mutation does not impair the demethylase activity but instead alters the binding to other proteins, perhaps involved into the recruitment of KDM5C to specific target promoters.

On the contrary, for the missense mutation p.R750W, the respective KDM5C protein band was severely reduced and the H3K4me3 band increased, with respect to the control. For this alteration, one explanation is that the substitution of the evolutionarily conserved arginine with a tryptophan residue in C5HC2-Zinc Finger domain of KDM5C [46] might cause a reduced protein stability followed by a protein degradation process operating as partial loss-of-function. We conclude that in NDD patient-derived cell lines, as a result of the KDM5C decrease (partial LoF) or the KDM5C absence (LoF), the global levels of H3K4me3 signal increase determine a perturbation of chromatin remodeling.

### 3.6. The Defective ARX-Dependent Transactivation of KDM5C Is Balanced by PHF8 or ZNF711 Overexpression

We previously showed that the loss of promoter stimulation of *KDM5C* caused by PHF8 mutant proteins was rescued by *ZNF711* coexpression; while the loss of KDM5C activity of mutant ZNF711 proteins was not compensated by WT *PHF8* coexpression [5]. Expanding this analysis, we cotransfected the JD-full-Luc construct with full-length Myc-tagged *ARX* (GenBank: NM_139058.2) WT and p.Ala105_Ala115dup and p.Ala148_Ala155dup mutant expressing vectors into SH-SY5Y cells (Figure 6C; Table 1). We observed that the coexpression of *ZNF711* with *PHF8* or individually led to normalization of the interfering defects of ARX-KDM5C interaction, associated with p.Ala105_Ala115dup and p.Ala148_Ala155dup mutations, increasing the luciferase quantity score back to the level obtained with the *ARX* WT construct (Figure 6C). This suggests that both the overexpression of ZNF711 and PHF8 might balance the impairment of the stimulation of *KDM5C* promoter in case of *ARX* mutations with partial loss-of-function.

## 4. Discussion

**Figure 7:**
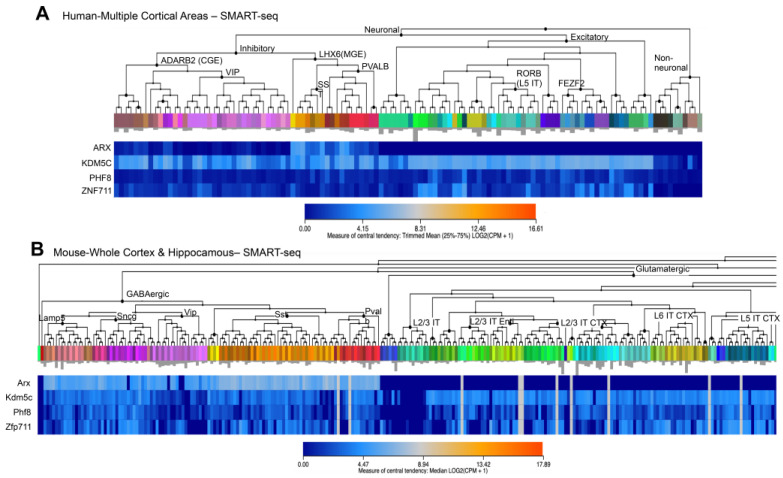
Heatmaps showing the expression profiles of *KDM5C/Kdm5c* and its regulatory genes *ARX/Arx*, *PHF8/Phf8*, and *ZNF711/Zfp711*. Data were obtained from Allen Brain Atlas/Transcriptomics Explorer in human (**A**) and mouse (**B**). Original images can be accessed freely at https://celltypes.brain-map.org/rnaseq/ (accessed on 10 July 2021).

Spatiotemporal gene expression differences across specific brain regions are guided by regulatory interactions through epigenetic modifications and transcriptional changes. Here, we reveal that a set of NDD genes, encoding the demethylases *KDM5C* and PHF8 and the transcription factors ARX and PHF8, is highly interconnected, showing a strongly coordinated expression pattern across neurogenesis and during neuronal differentiation of hiPSCs. These genes show a peculiar coexpression pattern in hippocampal and neocortical regions, with two intersected cardinal areas involved in flexible cognition and social behavior [48]. Moreover, three of them—*KDM5C*, *ARX*, and *PHF8*—show high expression levels at the timing of neuronal proliferation with a decrease during neuronal migration and synaptogenesis—two highly vulnerable processes underpinning neural circuit development [2]. By contrast, *ZNF711* shows an oscillatory trend with a very low expression level during the proliferation phase that increases, reaching a peak at the beginning of neuronal migration and synaptogenesis (Figure 7A). In embryonic and neonatal mouse brain, transcript and protein levels of *Kdm5c*, *Arx*, *Phf8*, and *Zfp711* show a similar framework: all four genes, and their relative proteins, are mainly coexpressed during the early stages of neurogenesis and neuronal migration with a gradual decrease until to the birth (Figure 7A).

Additional data based on FANTOM5 CAGE database reinforce the concept that this set of NDDs shows overlapping time-specific transcriptional modules. They operate during brain development—where they are involved in neuronal cell specification—and in adult brain areas—where they control neuronal plasticity and functioning. Noteworthily, CAGE analysis was successfully applied to characterize regulatory intersection among genes found mutated in other NDDs—such as Rett Syndrome—and build the atlas of TSS across major human cell-types and tissues [32,49]. The entry into the scene of the three regulating genes at various times of neurogenesis suggests that transcription of the epigenetic eraser KDM5C has a multi-level regulatory governance—particularly in human—capable of ensuring the correct dosage of KDM5C in response to specific stimuli at the proper time and in the proper tissue area or cell. Moreover, further investigation on the roles of these transcriptional modules in specific cell subtypes, such as glutamatergic neurons, GABAergic neurons and glia cells should be performed. To this end, single-cell RNA sequencing studies allow defining single cell coexpression landscapes and identifying targeted cells for potential treatments [50]. Remarkably, as reported in the Human Protein Atlas dataset (https://www.proteinatlas.org/ accessed on 10 July 2021), KDM5C and its three regulatory proteins ARX, ZNF711 and PHF8 are all expressed in the corpus callosum, in human, pig, and mouse (Table S4). Deciphering whether these subsets of NDD genes are mutually related to the development of callosal projection neurons (CPN), will shed light on the molecular mechanisms controlling the formation of the corpus callosum, a structure that connect the two cortical hemispheres. Noteworthily, one of the allelic disorders caused by ARX mutations is Proud syndrome, presenting as a main clinical features the agenesis of the corpus callosum [51]. Nevertheless, no gross alterations in the corpus callosum have been reported in patients mutated in KDM5C, ZNF711, and PHF8 [10,23,29,44,45,46].

Since defects in GABAergic neurons feature prominently in NDDs, another interesting aspect that requires deep investigation is that whether this subset of NDD genes is mutually involved in differentiation and functioning of GABAergic interneurons. By interrogating the Allen Brain Atlas RNAseq database, it is evident that they are mutually coexpressed in specific subsets of GABAergic neurons, both in human and mouse (Figure 7A,B; [52]). The direct involvement of ARX and KDM5C in GABAergic-related activities has been already reported [4,5,9,53]; while it is unknown the role of ZNF711 and PHF8 is unknown. Furthermore, 3D human organoids from patients could help to identify in detail the functional impact of each of them on the production of GABAergic neurons and whether they could alter the balance between GABAergic and glutamatergic neurons.

Another interesting observation is that the localization of *KDM5C* and its regulatory genes is conserved within synthenic blocks of X chromosomes in human and mouse. This conserved localization parallels their coexpression profiles in human and mouse nervous system, suggesting that the coordinated waves of gene expression during neurogenesis and in specific brain areas is controlled by conserved regulatory mechanisms. Indeed, binding sites for key lineage and/or tissue-specific transcription factors could be simultaneously recruited at the enhancers and active promoters of this set of X-linked genes. On the other hand, clustering of genes expressed during the early phases of brain development could be advantageous to assemble chromatin neighbourhoods with open conformation. Moreover, further studies are required to define whether they fall into the same topologically associating domain (TAD) and/or share common regulatory players.

Chromatin perturbations associated to mutations in neurodevelopmental genes damage vulnerable brain processes such as cognition development and neuronal homeostasis [2,3]. Notably, in patient-derived LCLs with mutations in *KDM5C*, *ARX*, *PHF8*, and *ZNF711*, we detected aberrant dosage of KDM5C and H3K4me3.

**Figure 8:**
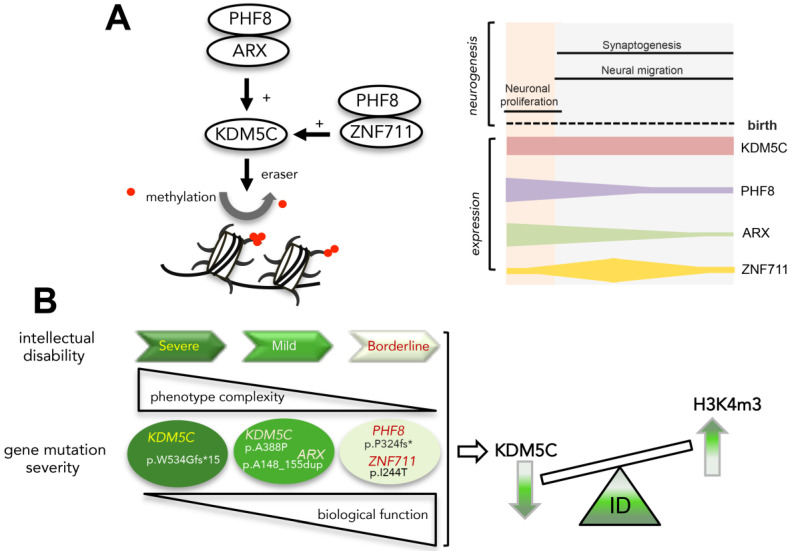
KDM5C is an epigenetic marker of ID severity. Regulatory governance of *KDM5C* transcription (**A**, **left panel**); and expression profiles of *KDM5C* and its regulatory genes during human neurogenesis (**A**, **right panel**); functional correlation between mutation severity and ID (**B**).

This suggests that the neurological diseases linked to this set of genes can be considered as X-linked chromatinopathies. Specifically, the aberrant levels of KDM5C and H3K4me3 in ID males mutated in *KDM5C*, *ZNF711, PHF8*, and *ARX* highlight a variable chromatin defect as a consequences of the type of disease mutation. This secondary disease hit could cause a relaxation in the chromatin conformation and compromise nervous system development. In reference to this, we observed a direct relationship between the severity of the KDM5C-H3K4me3 defect with the severity of cognition impairment. This feature highlights the value of KDM5C as a disease epigenetic marker to predict ID severity caused by mutations in its gene (direct effect) or in its regulator genes (indirect effect) (Figure 8B). Obviously, the KDM5C-H3K4m3 axis is not the solely damaged process, but other pathways can be altered as a result of mutations in genes involved in pleiotropic activities, such as *ARX*, *ZNF711*, or *PHF8*. We therefore propose a model explaining that the varying severity of ID, a common clinical core associated to the four disease genes, could be explained through the impact on KDM5C-H3K4me3 axis (Figure 8B). On the other hand, the identification of further regulatory elements of the KDM5C transcriptional axis (e.g., cofactors, interactors, non-coding RNAs, etc.) and molecular compounds capable of modulating its expression levels (e.g., epidrugs, small peptides, etc.) will contribute to deeply define how to counteract a KDM5C deficit in time-regulated windows of cognition development. In this direction are the recent findings on the functional interactions of KDM5C with lysine methyltransferase 2A (KMT2A; MIM 159555), responsible for Wiedemann-Steiner Syndrome (WDSTS; MIM:605130), proving that this eraser-writer pair acts in a mutually suppressive mode, ameliorating their associated disorders [9]. More interestingly, we have shown that in vitro and in vivo treatments with suberanilohydroxamic acid (SAHA) is a potent reversible pan-histone deacetylase (HDAC) inhibitor, is able to correct the KDM5C-H3K4me3 pathway [5].

A peculiar aspect of this highly connected regulatory axis is the capacity to rebalance the partial loss activity of expanded polyalanine tracts in ARX via overexpression of the coregulatory proteins ZNF711 or PHF8. Along the same line, we previously found that *ZNF711* overexpression is also capable of balancing the partial loss activity of PHF8 mutant proteins [5]. Collectively, these findings suggest that PHF8 and ZNF711 may act as gene modifiers of ARX-disease phenotypes; thus, variants in these NDD genes may contribute to the phenotype variability from West syndrome to Partington syndrome to mild ID caused by ARX polyalanine expansions. Even though more studies are needed to fully understand the peculiar activities of this intersected NDD pathway, we propose that the *KDM5C* transcription has a multi-level regulatory governance coupled to a positive feedback mechanism. In addition, because the expression profiles of KDM5C regulatory genes are mutually related to multiple cellular subtypes, as callosal projection neuron and interneurons, they could be involved in differentiation and functioning of varying neuronal cell types.

## 5. Conclusions

Our findings indicate that KDM5C is an epigenetic marker of developmental signatures through which we may estimate the functional impact of disease mutations on cognition processes and that it represents a convergent point for therapeutic intervention for a set of X-linked chromatinopathies.

## Figures and Tables

**Table 1:** List of disease mutations analyzed in this study.

Gene	cDNA Change	Protein Change	Mutation	Clinical Features	Reference
***ARX***	c.298_330dup33	p.Ala105_115dup	duplication	DEE1, severe ID	[4,18,42,43]
	c.429_452dup24	p.Ala148_155dup	duplication	mild ID	[4,18,42,43]
***KDM5C***	c.1162G>C	p.Ala388Pro	missense	ID, mild dysmorphism	[10]
	c.3864G>A	p.Trp1288X	nonsense	severe ID, spasticity, epilepsy, microcephaly	[10,44]
	c.1599delC	p.Trp534Glyfs*15	nonsense	severe ID, epilepsy, spasticity	[45]
	c.2248C>T	p.Arg750Trp	missense	severe ID, speech impairment	[46]
***PHF8***	c.1050_1061del12	p.Pro314fs*	nonsense	mild to borderline ID, cleft lip and cleft palate	[23]
***ZNF711***	c.731T>C	p.Ile244Thr	missense	mild to borderline ID, speech delay	[29]

ID, Intellectual disability; DEE1, Developmental epileptic encephalopathy-type 1.

## Data Availability

Publicly archived datasets analyzed: FANTOM5 database https://fantom.gsc.riken.jp, accessed on 18 June 2021; Human Brain Transcriptome Atlas https://hbatlas.org/hbtd/basicSearch.pl, accessed on 1 May 2021; Liber Institute for Brain development http://stemcell.libd.org/scb/, accessed on 1 May 2021; Allen Mouse Brain Atlas https://mouse.brain-map.org/, accessed on 1 May 2021; UCSC https://genome.ucsc.edu/; Cell Types Database https://celltypes.brain-map.org/rnaseq/, accessed on 1 May 2021.

## Supplementary Materials

The following are available online at https://www.mdpi.com/article/10.3390/genes12071088/s1, Figure S1: Gene expression levels of *KDM5C* and its regulatory genes throughout neuronal differentiation. Figure S2: Expression analysis of *Kdm5c*, *Arx, Phf8* and *Zfp711* in adult male mouse brain (P56). Figure S3: Zenbu genome browser views of gene locus for human *KDM5C*, *ARX*, *PHF8*, and *ZNF711.* Table S1: CAGE annotation from Zenbu database. Table S2: Human and murine expression data of CAGE obtained from Zenbu database. Table S3: Oligonucleotides used for transcript analysis. Table S4: RNA expression summary of KDM5C and its regulatory genes in the corpus callosum (from The Human Protein Atlas datasets; https://www.proteinatlas.org/, accessed on 1 May 2021)
