# Supplementary material for: Analysis of a Set of KDM5C Regulatory Genes Mutated in Neurodevelopmental Disorders Identifies Temporal Coexpression Brain Signatures
Source: Genes (Basel). 2021 Jul 18;12(7):1088. doi: 10.3390/genes12071088 (PMC8305412; doi:10.3390/genes12071088)
Supplement: Supplementary file 1 [file genes-12-01088-s001.zip › Figure S1-S3.pdf]

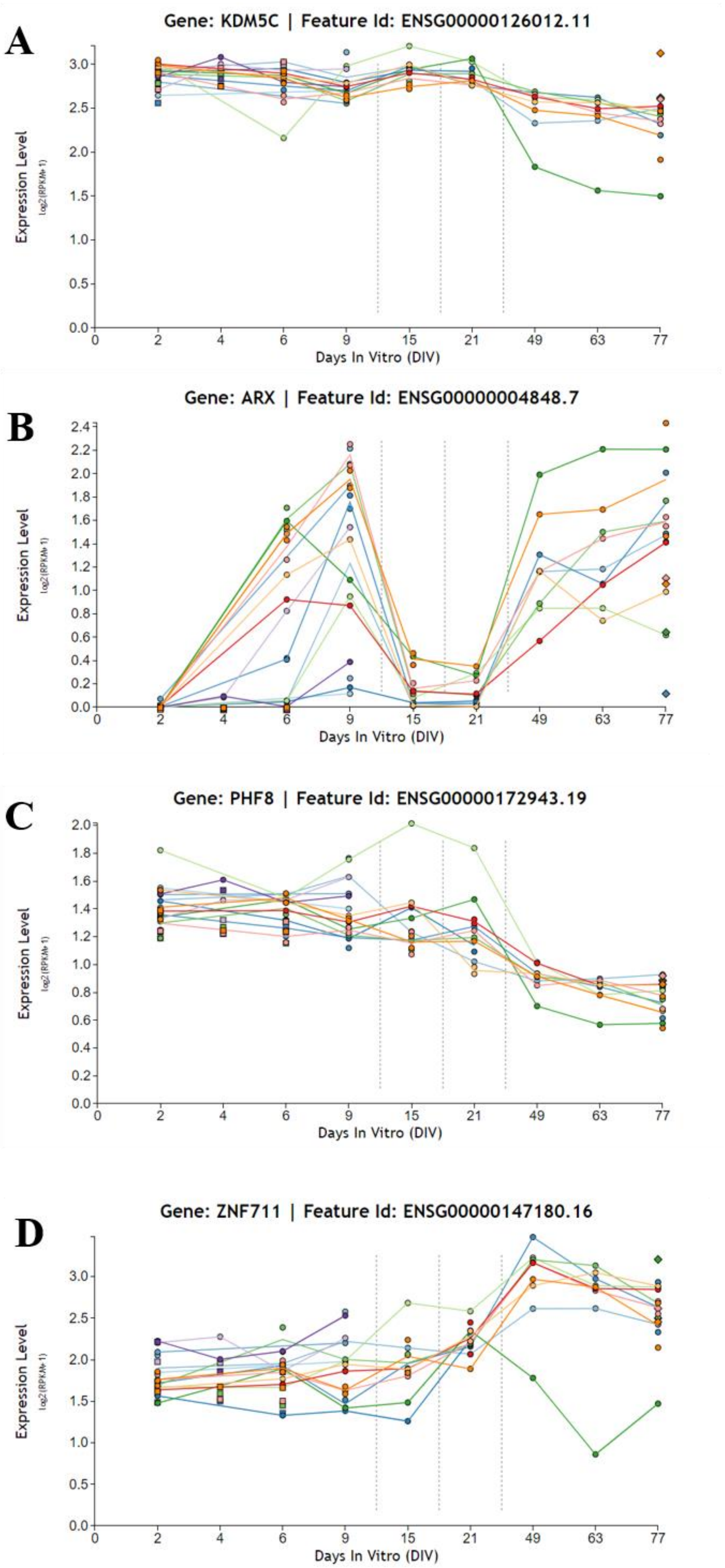
A
Gene: KDM5C | Feature Id: ENSG00000126012.11
Expression Level
log2(RPKM+1)
Days In Vitro (DIV)
B
Gene: ARX | Feature Id: ENSG00000004848.7
Expression Level
log2(RPKM+1)
Days In Vitro (DIV)
C
Gene: PHF8 | Feature Id: ENSG00000172943.19
Expression Level
log2(RPKM+1)
Days In Vitro (DIV)
D
Gene: ZNF711 | Feature Id: ENSG00000147180.16
Expression Level
log2(RPKM+1)
Days In Vitro (DIV)

Figure S1. Gene expression levels of *KDM5C* and its regulatory genes throughout neuronal differentiation. Using the Liber Institute for Brain development (LIBD) stem cell browser, created by Burke and colleagues [48], we report on gene expression changes during neuronal differentiation of *KDM5C* **(A)**, *ARX* **(B)**, *PHF8* **(C)** and *ZNF711* **(D)**. The square points at days 2, 4 and 6 in vitro (DIV) are the self-renewal condition, the circular points at 2, 4, 6 and 9 DIV are the accelerated dorsal condition, the circular points at 15 DIV are the NPCs, the circular points at 21 DIV are rosettes, the circular points at 49, 63, and 77 DIV are neurons grown with rat astrocytes and the diamond points at 77 DIV are pure neuronal cells. Each graph has the f-statistic and q-value statistic for the differential expression over the entirely differentiation process generated from the LIBD stem cell browser available at http://stemcell.libd.org/scb/ .

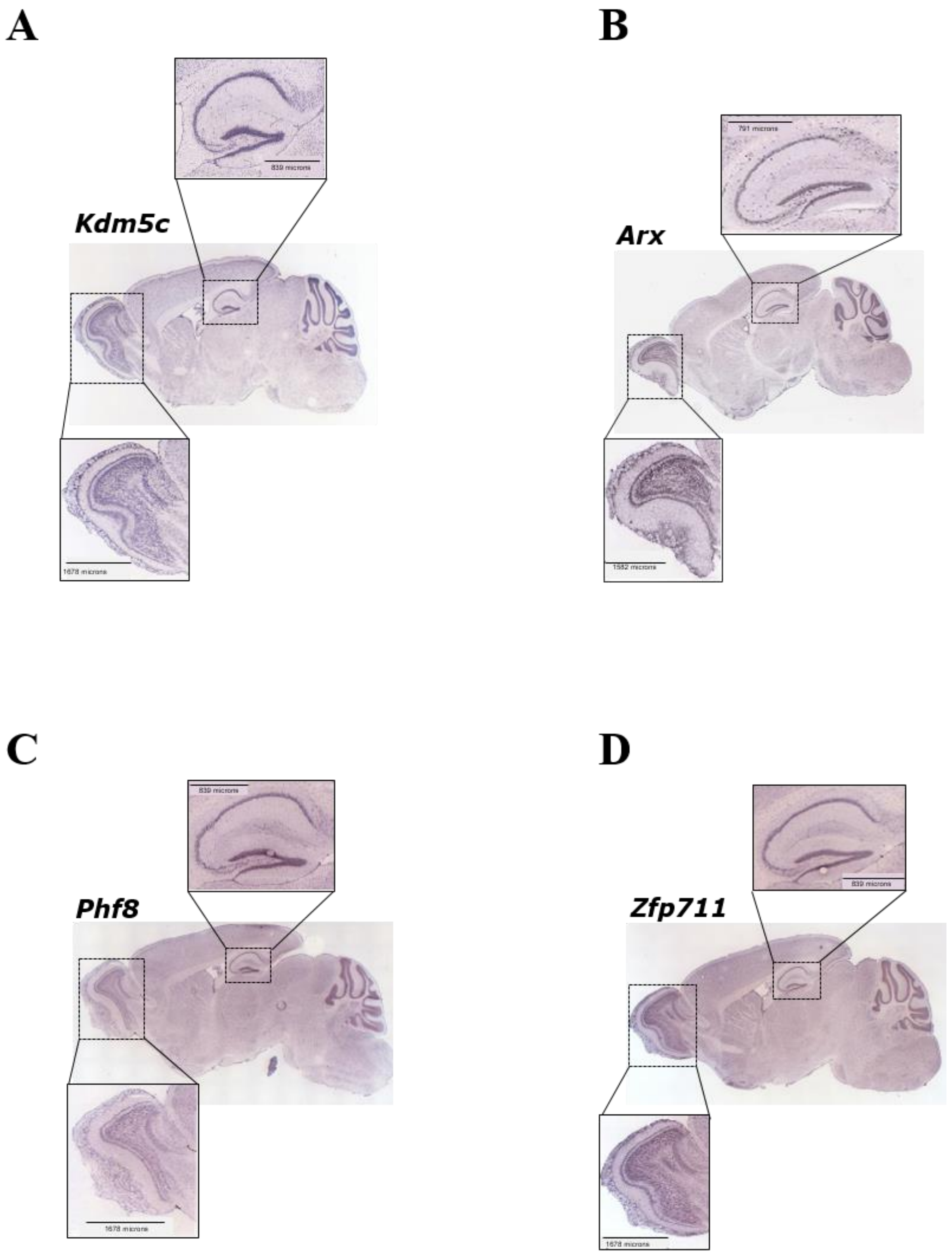

Figure S2. Expression analysis of *Kdm5c, Arx, Phf8* and *Zfp711* in adult male mice brain (P56). *in situ* hybridization images were obtained from Allen institute for Brain Science. Images showed the staining for each gene in sagittal section of mice brain. Zoomed views for hippocampus and olfactory bulbs are shown.

Original images can be accessed freely on https://mouse.brain-map.org/ (*Kdm5c*: https://mouse.brain-map.org/gene/show/20353; *Arx*: https://mouse.brain-map.org/ experiment/ show /100144536; *Phf8*: https://mouse.brain-map.org/gene/show/107530; and *Zfp711*: https://mouse.brain-map.org/gene/show/89415).

A

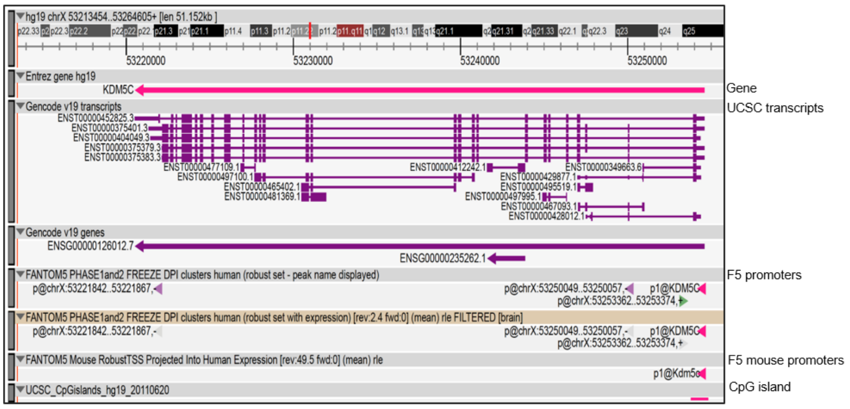
KDM5C
Gene
UCSC transcripts
F5 promoters
F5 mouse promoters
CpG island

B

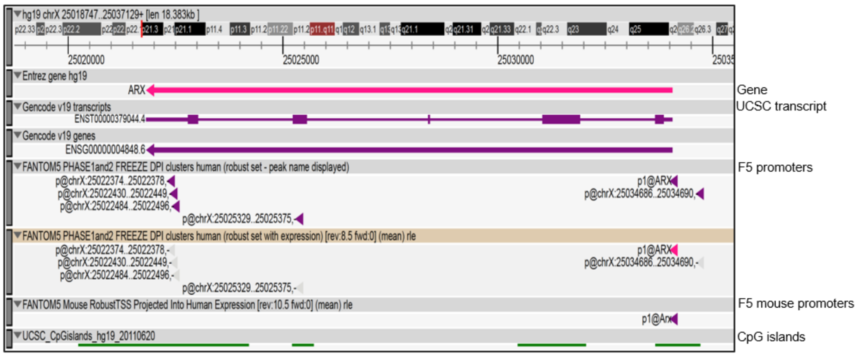
ARX
Gene
UCSC transcript
F5 promoters
F5 mouse promoters
CpG islands

C

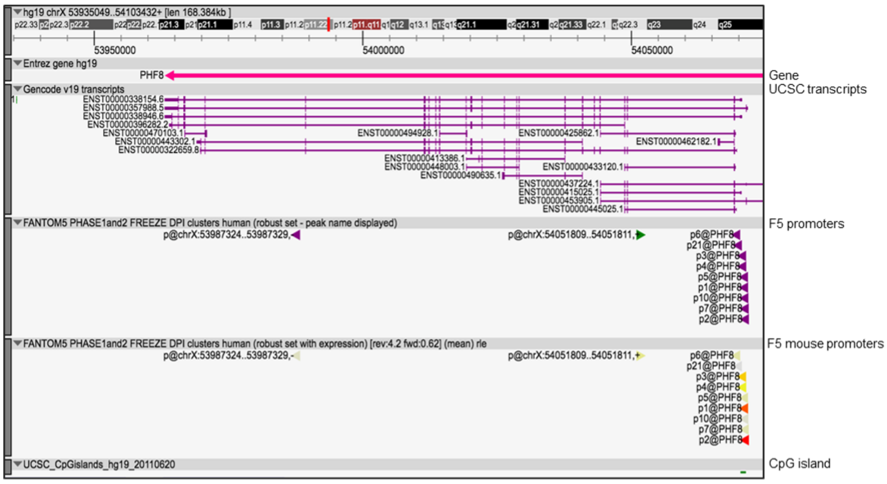
PHF8
Gene
UCSC transcripts
F5 promoters
F5 mouse promoters
CpG island

D

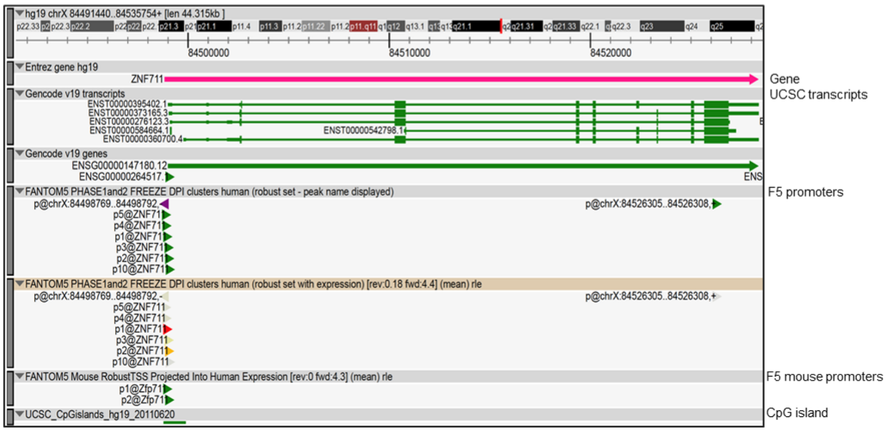
ZNF711
Gene
UCSC transcripts
F5 promoters
F5 mouse promoters
CpG island

Figure S3. Zenbu genome browser views of gene locus for human *KDM5C* **(A)**, *ARX* **(B)**, *PHF8* **(C)** and *ZNF711* **(D)**. Genes and transcripts are color-coded according to their orientation in the genome (+ strand, green; - strand, purple). Green arrows highlight tags of antisense orientation. Annotated UCSC transcripts are shown, with exon (thick lines) and intron (thin lines) boundaries. FANTOM5 promoters (robust DPI and robust DPI with expression) are indicated as small arrowheads, color-coded according to the direction of transcription. CpG islands are indicated as thick lines with color-coded according to the direction of transcription.
