# Supplementary material for: Analysis of a Set of KDM5C Regulatory Genes Mutated in Neurodevelopmental Disorders Identifies Temporal Coexpression Brain Signatures
Source: Genes (Basel). 2021 Jul 18;12(7):1088. doi: 10.3390/genes12071088 (PMC8305412; doi:10.3390/genes12071088)
Supplement: Supplementary file 1 [file genes-12-01088-s001.zip › Table S3.pdf]

Supplementary Table 3 Oligonucleotides used for transcript analysis

Gene	Sequence
mArx	*F 5'*-GCTGGGTCTGAGCACTTTTC-3'
	R 5'-GGTGTGGGCTGTCTCAGG-3'
mSyn1	*F 5'*-CTGGGCAAAATACTTCAAAGGG-3'
	R 5'-GTCCACAGAGAATCCACCATTG-3'
mPhf8	*F 5'*-ACTGCCCTAACTGTGAGGTC-3'
	R 5'-CAAGACAAGGATAGGCACGC-3'
mZfp711	*F 5'*-GTGATTCAAGCAGGTGGTGG-3'
	R 5'-TTTTCAATGGCGTGTTCCCC-3'
mKdm5C	*F 5'*-TTCCTTGCTACGCTCTCACTATGA-3'
	R 5'-TCAAATGGGCGTGTGTTACAC-3'
hKDM5C	*F 5'*-CTCCTTGCTACGCTCCCACTACGA-3'
	R 5'-TCAAATGGACGTGTGTTACAC-3'
18S	*F 5'*-CAATCCGCGGCGCTTATTCCCAT-3'
	R 5'-AATCTGTCAATCCTGTCCGT-3'
ACTB	*F 5'*-CATGTACGTTGCTATCCAGGC-3'
	R 5'-CTCCTTAATGTCACGCACGAT-3'

F = Forward; *R* = Reverse. m= mouse; h= human
