# Supplementary material for: Analysis of a Set of KDM5C Regulatory Genes Mutated in Neurodevelopmental Disorders Identifies Temporal Coexpression Brain Signatures
Source: Genes (Basel). 2021 Jul 18;12(7):1088. doi: 10.3390/genes12071088 (PMC8305412; doi:10.3390/genes12071088)
Supplement: Supplementary file 1 [file genes-12-01088-s001.zip › Table S4.pdf]

Table S4. RNA expression summary of KDM5C and its regulatory genes in the corpus callosum (from The Human Protein Atlas datasets; https://www.proteinatlas.org/)

**Gene**	**FANTOM5*** **FF:10042-101F6** (scaled tags/million)	**Pig RNA-seq**** (pTPM; N=4)	**Mouse RNA-seq***** (pTPM; N=3)
ARX	10.8	4.0	7.4
KDM5C	65.7	25.8	12.8
PHF8	30.6	18.0	6.9
ZNF711	9.5	7.8	2.1

* RNA expression obtained through Cap Analysis of Gene Expression (CAGE) generated by FANTOM5 project is reported as scaled tags per million;
** RNA-seq data obtained from pig brain reported as transcripts per million protein coding genes (pTPM);
*** RNA-seq data obtained from mouse brain reported as pTPM
